# Immune responses of cattle vaccinated by various routes with *Mycobacterium bovis* Bacillus Calmette-Guérin (BCG)

**DOI:** 10.1186/s12917-024-04452-7

**Published:** 2025-01-15

**Authors:** Mitchell V. Palmer, Soyoun Hwang, Carly Kanipe, Ellie J. Putz, Luis Guilherme Virgilio Fernandes, Anna Didkowska, Paola M. Boggiatto

**Affiliations:** 1 Bacterial Diseases of Livestock Research Unit, National Animal Disease Center, Agricultural Research Service, 1920 Dayton Ave, Ames, IA 50010 USA; 2 Center for Veterinary Biologics, Animal and Plant Health Inspection Service, USDA, 1920 Dayton Ave, Ames, IA 50010 USA; 3 Immunobiology Graduate Program, College of Veterinary Medicine, Iowa State University, 1800 Christensen Drive, Ames, IA 50010 USA; 4 Oak Ridge Institute for Science and Education, 1299 Bethel Valley Road, Oak Ridge, TN 37830 USA; 5 Department of Food Hygiene and Public Health Protection, Institute of Veterinary Medicine, Warsaw University of Life Sciences (SGGW), Nowoursynowska 159, Warsaw, 02-776 Poland

**Keywords:** BCG, Bovine, Lyophilization, Mycobacteria, Oral, Tuberculosis, Vaccination

## Abstract

**Background:**

*Mycobacterium bovis* BCG is the human tuberculosis vaccine and is the oldest vaccine still in use today with over 4 billion people vaccinated since 1921. The BCG vaccine has also been investigated experimentally in cattle and wildlife by various routes including oral and parenteral. Thus far, oral vaccination studies of cattle have involved liquid BCG or liquid BCG incorporated into a lipid matrix. Lyophilization is an established technique used for stabilizing bioproducts such as vaccines.

**Methods:**

In the current study, cattle were vaccinated in two phases. In each phase, cattle were divided into three groups. Group 1 received BCG injected SQ, Group 2 received liquid BCG delivered to the posterior oral cavity, Group 3 orally consumed lyophilized BCG contained within a gelatin capsule placed within a small amount of a commercial alfalfa product.

**Results:**

No vaccinated cattle were positive by an interferon gamma release assay. All but 4 animals were negative by tuberculin skin testing prior to vaccination: the 4 non-negative animals being categorized as suspects. Sixteen weeks post-vaccination all but 1 animal was negative, it being categorized as a suspect. An in vitro antigen stimulation assay and flow cytometry were used to detect antigen-specific CD4, CD8 and γδ T cell responses following vaccination. Oral vaccination of animals with lyophilized BCG did not result in any increases in the frequency of CD4, CD8 or γδ T cell proliferative or IFN-γ responses at any of the time points analyzed in either phase 1 or 2. In contrast, vaccination with BCG SQ and liquid BCG delivered to the posterior pharynx, resulted in an increase in the frequency of proliferating and IFN-γ-producing CD4 T cells with peak responses at 9–12 weeks post-vaccination. Similar to oral lyophilized BCG vaccinated animals, we did not observe any significant increases in the frequency of CD8 and γδ T cell proliferative and IFN-γ responses following SQ or oral liquid vaccinated animals.

**Conclusions:**

These data would suggest that vaccination with oral lyophilized BCG does not induce a measurable, antigen-specific cell mediated responses in the periphery, when compared to BCG administered SQ or liquid BCG administered via the oral route. However, vaccination with either SQ or liquid BCG delivered to the posterior pharynx does induce measurable CD4 T cell responses in the periphery.

**Supplementary Information:**

The online version contains supplementary material available at 10.1186/s12917-024-04452-7.

## Background

Tuberculosis (tb) in humans is generally caused by *Mycobacterium tuberculosis*. The only approved tb vaccine is the attenuated strain of *Mycobacterium bovis* known as bacillus Calmette-Guérin (BCG), named for Albert Calmette and Camille Guérin, two French scientists at the Pasteur Institute that developed the strain [1]. First used in humans in 1921, BCG vaccines are the oldest vaccines still in use today. Furthermore, with over 4 billion people vaccinated in more than 180 countries, it is the safest and most widely used vaccine in history [2].

Nevertheless, protective immunity in adults is highly variable, ranging from 0 to 80% depending on the study [3]. In spite of wide-ranging estimates of efficacy, in infants BCG has proven beneficial and highly cost-effective in protecting children from tuberculous meningitis [4, 5]. The use of BCG vaccination in cattle has been extensively reviewed [6]. Like BCG vaccination in humans, efficacy varies between studies. Additionally, routes of vaccination have been investigated including parenteral and oral [7, 8]. In some previous cattle studies, orally delivered BCG was incorporated into a lipid matrix [9]. This lipid-based matrix-BCG was also used in mice, brushtail possums (*Trichosurus vulpecula*), and white-tailed deer (*Odocoileus virginianus*), resulting in decreased disease severity in experimental challenge studies [10, 11].

The lipid-based matrix-BCG was originally designed for use in monogastric species as the lipid is designed to protect BCG from degradation by gastric secretions allowing uptake of live BCG across the intestinal wall [12].

Oral vaccines offer several advantages for the immunization of wildlife and livestock including ease of use and potential to stimulate mucosal and systemic immune responses.

For wildlife, oral vaccines are the most practical and cost-effective means of vaccination. Success has been seen with the use of oral rabies vaccines used to vaccinate wildlife such as foxes, raccoons, coyotes, and skunks [13, 14]. Similarly, an oral vaccine against *Yersinia pestis* infection has been used in populations of endangered black-footed ferrets [15].

The objective of the present study was to use calves to investigate the immune response of animals vaccinated orally with *M. bovis* BCG in either liquid or lyophilized form.

Lyophilization is a well-established technique used in the pharmaceutical industry for stabilizing bioproducts such as vaccines [16]. Lyophilization can reduce the need for a cold chain making vaccines available for use in lower socioeconomic regions, as well as potentially advantageous for stabilizing a wildlife bait vaccine. Additionally, lyophilized vaccines can easily be incorporated with various substrates suitable for targeting various species.

## Results

### Interferon gamma release assay (IGRA) responses following BCG vaccination

**Fig. 1:**
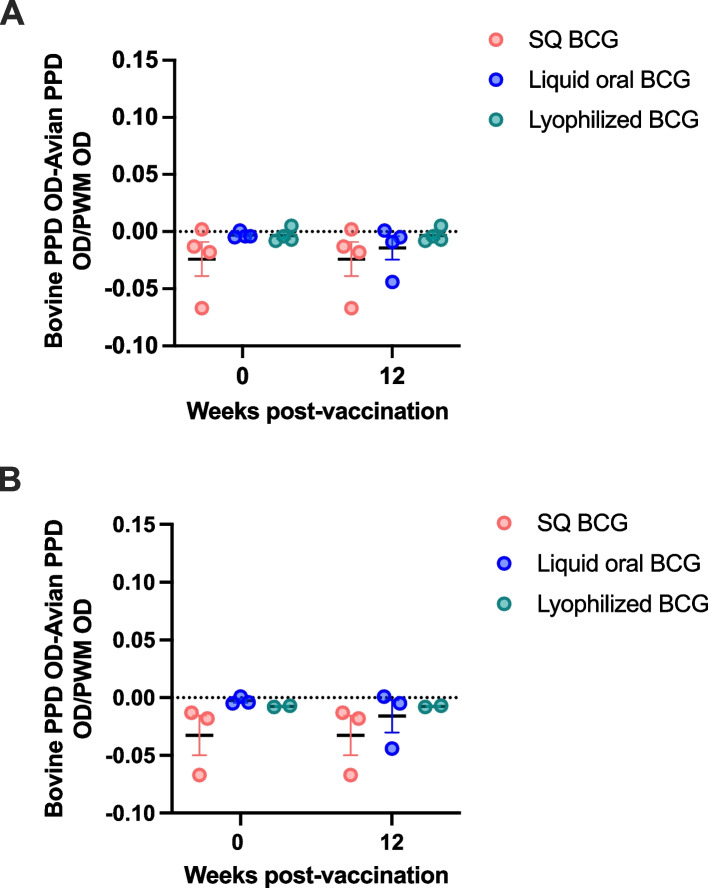
Interferon gramma release assay (IGRA) responses of animals vaccinated with *M. bovis* BCG via different routes of administration. **A** Phase 1, **B** Phase 2. Data are presented as ΔOD with ≥ 0.1 used as cut-off

For initial assessment of cellular effector responses induced by vaccination with BCG via the different routes, we performed an IGRA. Experimental vaccination was carried out in two independent phases, utilizing two cohorts of age-matched cattle. Prior to and following vaccination with subcutaneous (SQ), orally delivered liquid, or oral lyophilized BCG, blood samples were collected and assessed for IFN-γ release prior to and at 12 weeks following vaccination. Regardless of the route of vaccination, we did not observe IGRA positive animals in either phase 1 (Fig. 1A) or phase 2 (Fig. 1B). These data would suggest that at the timepoints analyzed, these vaccination platforms did not induce measurable IGRA responses that would interfere with routine IGRA diagnostic testing.

### Tuberculin skin testing results

Prior to vaccination 9/11 animals in phase 1 and 7/9 animals in phase 2 were considered negative by the comparative cervical test (CCT) (Supplemental Table 2). Of the non-negative results, there were 2 suspects in phase 1, and 2 suspects in phase 2. Sixteen weeks postvaccination, 10/11 animals in phase 1 and 9/9 animals in phase 2 were considered negative. One animal in phase 1, which was categorized as negative prior to vaccination was considered a suspect post-vaccination. All animals categorized as suspects prior to vaccination were categorized as negative when tested 16 weeks post-vaccination.

### Antigen-specific PBMC responses following vaccination

**Fig. 2:**
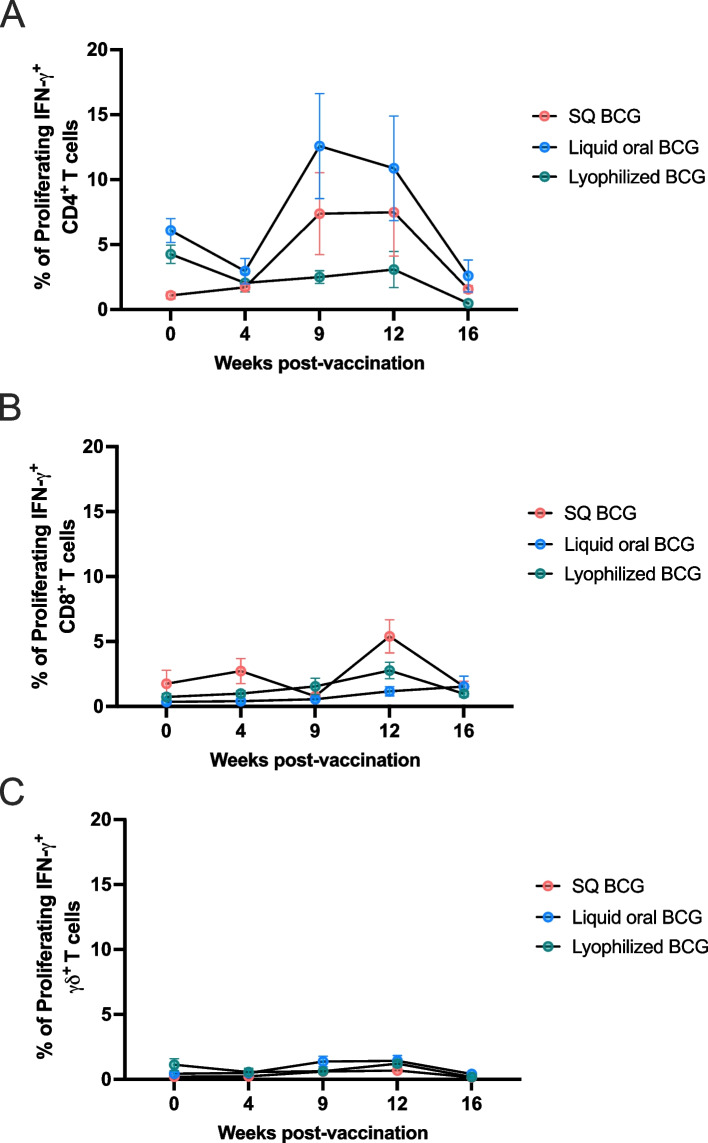
Phase 1; Cell mediated proliferative and IFN-γ responses in cattle following BCG vaccination via different administration routes. Oral vaccination of animals with lyophilized BCG (green circles) did not result in any increases in the frequency of CD4 (**A**), CD8 (**B**) or γδ (**C**) T cell proliferative or IFN-γ responses at any of the time points analyzed. Vaccination with BCG SQ (red circles) and orally delivered liquid BCG (blue circles), resulted in increases in the frequency of proliferating and IFN-γ-producing CD4 T cells (**A**). Responses peak at approximately 9 weeks post-vaccination and demonstrate a slow decay to 16 weeks post-vaccination. There were no significant increases in the frequency of CD8 (**B**) and γδ (**C**) T cell proliferative and IFN-γ responses following SQ or orally delivered liquid vaccinated animals

To further assess the cellular immune responses elicited by vaccination via these different routes, we performed in vitro recall response assays to measure memory T cell responses to vaccination via proliferation and IFN-γ production. An in vitro antigen stimulation assay and flow cytometry were used to detect antigen-specific CD4, CD8 and γδ T cell responses following vaccination. Flow cytometry gating strategy and representative dot plots for CD4, CD8, γδ, IFNγ and proliferation are shown in Supplemental Fig. 1. In phase 1, vaccination of animals with oral lyophilized BCG did not result in any increases in the frequency of CD4 (Fig. 2A), CD8 (Fig. 2B) or γδ (Fig. 2C) T cell proliferative or IFN-γ responses at any of the time points analyzed. In contrast, vaccination with BCG SQ and orally delivered liquid BCG, resulted in an increase in the frequency of proliferating and IFN-γ-producing CD4 T cells over time (*p* = 0.0028) (Fig. 2A). Both responses appear to peak at approximately 9 weeks post-vaccination and demonstrate a slow decay to 16 weeks post-vaccination. Similar to oral lyophilized BCG vaccinated animals, we did not observe any significant increases in the frequency of CD8 (Fig. 2B) and γδ (Fig. 2C) T cell proliferative and IFN-γ responses following SQ or oral liquid vaccinated animals.

**Fig. 3:**
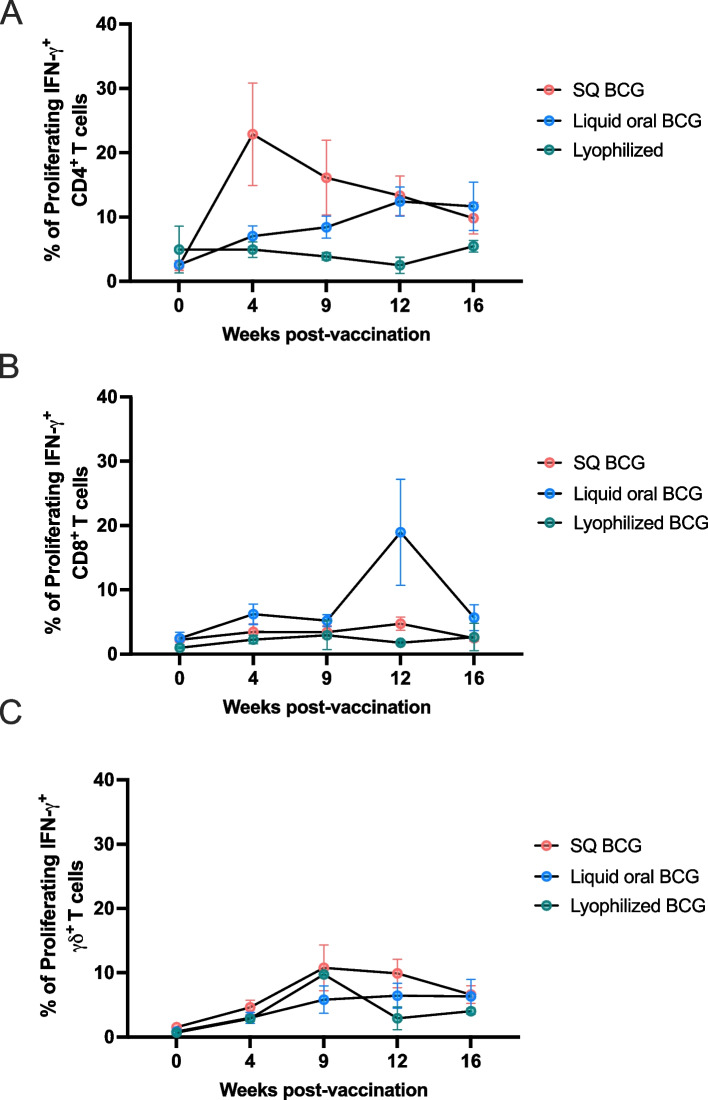
Phase 2; CD4, CD8, and γδ T cell proliferative and IFN-γ responses following BCG vaccination via different administration routes. **A** Oral vaccination of animals with lyophilized BCG (green circles) did not result in any increases in the frequency of CD4, CD8 (**B**) or γδ (**C**) T cell proliferative or IFN- responses at any of the time points analyzed. Vaccination with BCG SQ (red circles) and orally delivered liquid BCG (blue circles), resulted in increases in the frequency of proliferating and IFN-γ-producing CD4 T cells. It is worth noting that the kinetics of the CD4 T cell response observed in phase 2 were different than those observed in phase 1. SQ vaccinated animals (red circles) showed peak responses at 4 weeks post-vaccination, slowly decaying out to 16 weeks post-vaccination (**A**). Animals vaccinated with orally delivered liquid BCG (blue circles) showed a steady increase in the frequency of antigen-specific CD4 T cells over the course of the study, with a modest peak in the response not observed until 12 weeks post-vaccination (**A**). CD8 (**B**) and γδ (**C**) T cell responses following SQ or orally delivered liquid BCG vaccination did not result in any significant increases in the frequency of proliferating and IFN-γ-producing cells

Consistent with the findings from the phase 1 vaccination study, in phase 2, we did not observe any increases in the frequency of antigen-specific proliferating or IFN-γ responses following vaccination with oral lyophilized BCG for CD4 (Fig. 3A), CD8 (Fig. 3B) or γδ (Fig. 3C) T cells at any of the timepoints analyzed. Vaccination with BCG SQ or via orally delivered liquid BCG again resulted in an increase in the frequency of circulating antigen-specific, proliferative and IFN-γ producing CD4 T cell responses over time (Fig. 3A). However, these increases over time were not statistically significant (*p* = 0.15). It is worth noting that the kinetics of the CD4 T cell response observed in phase 2 was different than that observed in phase 1. In phase 1, peak responses were seen between 9–12 weeks post-vaccination (Fig. 2A). In comparison, in phase 2, animals vaccinated with BCG SQ showed a peak response at 4 weeks post-vaccination, slowly decaying out to 16 weeks post-vaccination (Fig. 3A), while animals vaccinated with oral liquid BCG showed a steady increase in the frequency of antigen-specific CD4 T cells over the course of the study, with a modest peak in the response not observed until 12 weeks post-vaccination (Fig. 3A).

Analysis of CD8 (Fig. 3B) and γδ (Fig. 3C) T cell responses in phase 2 following SQ or orally delivered liquid BCG vaccination did not result in any significant increases in the frequency of proliferating and IFN-γ-producing cells. Altogether, these data would suggest that oral vaccination with lyophilized BCG does not induce measurable, antigen-specific cell mediated responses in the periphery, when compared to BCG administered SQ or orally delivered liquid BCG. However, vaccination with either SQ or orally delivered liquid BCG does induce measurable CD4 T cell responses in the periphery, albeit with a certain degree of variability between phases.

**Fig. 4:**
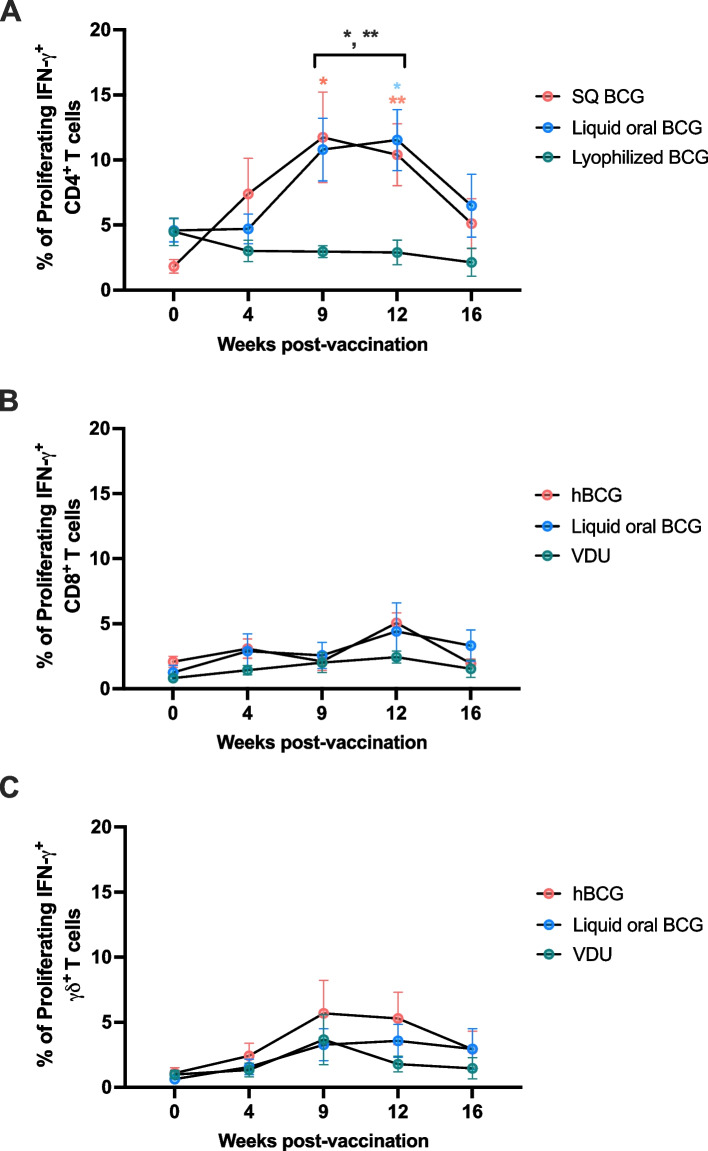
Phases 1 and 2 combined CD4, CD8, and γδ T cell proliferative and IFN-γ responses in cattle following BCG vaccination via different administration routes. Lyophilized oral BCG does not induce measurable peripheral CD4 T cell responses (**A**), in contrast to SQ or oral liquid BCG. The combined data demonstrate that measurable CD8 (**B**) or γδ (**C**) T cell responses were not observed following vaccination with any of the vaccination routes at any time points examined. Statistically significant differences within vaccinated groups, indicated by red (SQ BCG) or blue (liquid oral BCG) asterisks. Statistical differences between vaccinated groups, indicated by black asterisk. * denotes p ≤ 0.05 and ** denotes p ≤ 0.001

We additionally evaluated and statistically analyzed the combined CD4, CD8 and γδ T cell responses of phase 1 and phase 2 (Fig. 4). As shown above, oral lyophilized BCG did not induce measurable peripheral CD4 T cell responses over the timepoints analyzed, while SQ or orally delivered liquid BCG resulted in significant (*p* = 0.0046) increases of proliferative responses over time. Specifically, in SQ BCG vaccinated animals we observed a significant increase in CD4 T cell proliferative responses at 9- and 12-weeks post-vaccination (*p* = 0.029 and *p* = 0.092, respectively) as compared to pre-vaccination frequencies. Similarly, liquid oral BCG vaccinated animals show a significant increase (*p* = 0.0418) in CD4 responses at 12 weeks post vaccination as compared to day 0. Furthermore, SQ BCG and liquid oral BCG were statistically different (SQ BCG *p* = 0.039 and 0.016; liquid BCG *p* = 0.016 and 0.0092) from lyophilized BCG vaccinated animals at both 9- and 12- weeks post-vaccination. The combined data analysis also demonstrated that measurable CD8 (Fig. 2B) or γδ (Fig. 2C) T cell responses were not observed following vaccination with any of the vaccination routes. While the distinct kinetics of the CD4 T cell response observed between the two experimental phases raises important questions regarding the variability of cellular immune responses to BCG, the combined CD4 T cell data, support the same observation as when the data are presented separately: lyophilized BCG does not result in measurable peripheral cellular responses following vaccination.

### Stability of lyophilized M. bovis* BCG*

To ensure that the process of lyophilization did not have a deleterious effect on the viability of the BCG vaccine, pre- and post-lyophilization counts were determined. Pre- and post-lyophilization counts were found to be 6.4 × 10^7^ CFU/ml and 1.3 × 10^7^ CFU/ml respectively (Supplemental Table 3 and 4), demonstrating a minimal effect of lyophilization on BCG viability. Moreover, although storage at 33.8 °C and 25 °C resulted in losses of approximately 2-3 logs after 2 months and 12 months, respectively, storage at −4 °C resulted in an approximate 1 log loss in CFU/ml and storage at −20 °C resulted in no loss even after 12 months of storage (Supplemental Tables 3 and 4).

### RT-qPCR cytokine expression

**Fig. 5:**
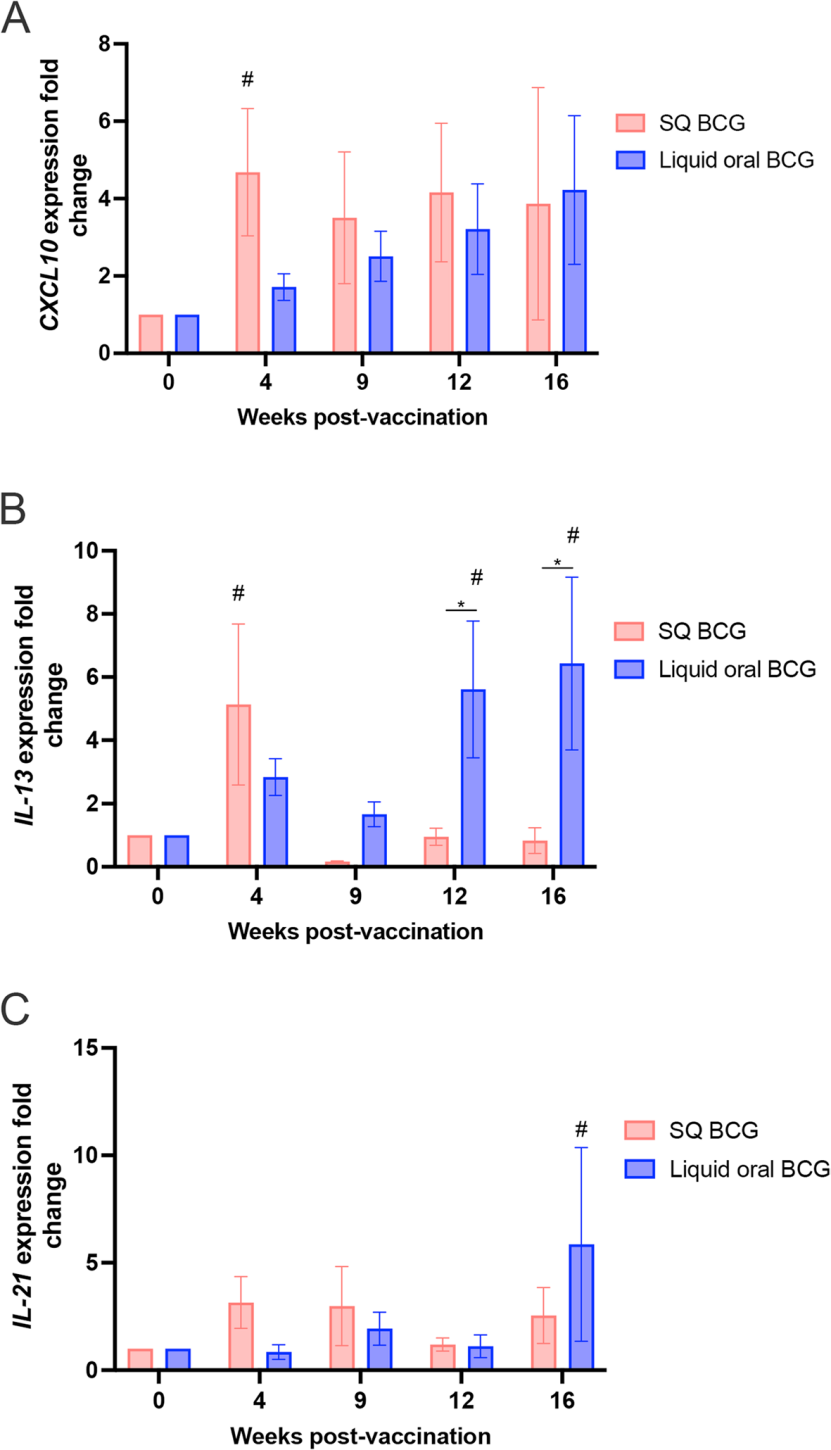
Cytokine gene expression profile following vaccination with BCG via different routes. **A** Compared to pre-vaccination levels, there is an increase in *CXCL10* expression in vaccinated animals, with peak fold-change observed at 4 weeks post-vaccination for SQ vaccinated animals (red). Expression of *CXCL10* remains elevated to the end of the study. In orally delivered liquid BCG vaccinated animals (blue), *CXCL10* expression showed a slow, and steady increase over time. **B** Compared to pre-vaccination levels, *IL-13* expression peaked at 4 weeks post-vaccination in SQ vaccinated animals, and quickly declined by 8 weeks postvaccination. In animals receiving orally delivered liquid BCG there was a delayed response, with a progressive increase in expression of *IL-13,* peaking at 16 weeks post-vaccination. **C** Compared to pre-vaccination levels, IL-21 expression was relatively low in SQ vaccinated animals, but peaked at 16 weeks post-vaccination in orally delivered liquid BCG vaccinated animals. # = *p* ≤ 0.05

Having demonstrated that lyophilized BCG did not induce cellular mediated responses following vaccination, we sought to further characterize the immune responses elicited by vaccination with SQ and orally delivered liquid BCG. To do this, we opted to characterize cytokines upregulated following overnight in vitro antigen stimulation of whole blood samples from SQ and orally delivered liquid BCG vaccinated animals from phase 2. We measured the expression of *CXCL10, IL-13 and IL-21* via RT-qPCR. Consistent with the antigen-specific CD4 T cell response data observed in Fig. 4A, we observed an increase in *CXCL10* expression in vaccinated animals, with peak fold-change in expression observed at 4 weeks post-vaccination for SQ vaccinated animals (Fig. 5A). Expression of *CXCL10* remained elevated at all timepoints analyzed. In orally delivered liquid BCG vaccinated animals, *CXCL10* expression showed a slow, and steady increase over time (Fig. 5A). These data are also consistent with the steady increase in CD4 T cell responses following oral liquid BCG vaccination.

We also assessed *IL-13* expression, a cytokine commonly associated with T helper 2, or humoral-mediated responses [17]. Following SQ vaccination, *IL-13* expression peaked at 4 weeks post-vaccination, and quickly declined by 8 weeks post-vaccination (Fig. 5B). In contrast, orally delivered liquid BCG vaccinated animals had a delayed response, with a progressive increase in expression of *IL-13,* peaking at 16 weeks post-vaccination (Fig. 5B). Vaccination with either SQ or orally delivered liquid BCG also resulted in a mild increase in expression of *IL-21* (Fig. 5C), again, with differing kinetics between vaccination routes. Animals vaccinated via the SQ route remained relatively low, showing a slight increase at 4–8 weeks post-vaccination. In contrast, orally delivered liquid BCG vaccinates spiked at 16 weeks post-vaccination but were otherwise relatively low in their *IL-21* expression.

## Discussion

The earliest vaccinations of humans with BCG were done in infants using an oral suspension [18], which became common practice. In the ensuing decades, as the vaccination program expanded to include adults, oral administration was gradually replaced by intradermal or percutaneous routes of administration [19, 20], although in some regions such as Brazil, oral vaccination with BCG Moreau continued until 1974 [19, 20]. Cumulatively, between 1921 and 1974 BCG was administered orally to millions of individuals [21].

For tb, the optimal route of administration should direct vaccine to sites of mucosal immune activation. Oral delivery of vaccines allows antigens to interact with the mucosa-associated lymphoid tissue (MALT) and can induce both mucosal and systemic immune responses [22]. The MALT includes lymphoid tissues in the nasopharynx, gastrointestinal tract and the cervical and vaginal mucosa [23]. In cattle and many other species, several oropharyngeal lymphoid tissues form Waldeyer’s ring composed of the palatine tonsils, pharyngeal tonsils, tubal tonsils and lingual tonsils [24, 25]. Tonsils have been described as the “Peyer’s patches” of the upper respiratory tract where they are active in antigen uptake and processing leading to the generation of antigen specific T and B cell responses [26]. Bovine palatine and pharyngeal tonsils have been shown to contain many of the same immune cell types as Peyer’s patches, including cells consistent with M-cells, and have been shown to internalize latex beads in ex vivo tonsil organ cultures [27, 28]. Due to their position at the junction of the nasopharynx and oropharynx, tonsils are well positioned to sample antigens entering via either the nasal cavity or oral cavity. Human tonsils and pharyngeal tonsils (adenoids) are shown to have an important role in mucosal immunity in the upper respiratory tract [29]. Sampling of orally administered BCG by the tonsils may be important in the generation of an effective immune response as has been demonstrated for orally delivered rabies and classical swine fever vaccines [30, 31]. Free liquid deposited in the posterior pharynx may more effectively expose tonsillar tissue to BCG than lyophilized BCG, thereby, enhancing the mucosal immune response. This possible difference in tonsillar exposure may contribute to the variation seen in immune responses between animals receiving orally delivered liquid vaccine and those receiving oral lyophilized BCG. Although beyond the scope of this paper, vaccine efficacy studies evaluating various routes of vaccination including intranasal would be beneficial.

Immune control of *Mycobacterium* infections relies on a T helper 1 (Th1) immune response driven by CD4 T cells and functional proliferation and production of IFN-γ [32–34]. Although lyophilization is commonly used in vaccine manufacturing, in the current study administration of a lyophilized BCG did not induce this critical T cell proliferative response, in contrast to orally delivered liquid BCG or BCG administered SQ. Lyophilization of vaccine affords many advantages. Liquid vaccines generally require storage at −20 °C and have a limited shelf-life after thawing. Lyophilized vaccine can often be stored at −4 °C or −20 °C for extended periods of time with little loss of viability, as demonstrated in the current study. Lyophilized vaccine can be easily encapsulated providing many options for vaccine delivery preparations targeting wildlife species such as bait vaccines. Despite the many advantages of lyophilized vaccines, in the current study vaccination using lyophilized BCG via the oral route did not induce a T cell proliferative response like that seen with orally delivered liquid or SQ administered BCG. The reasons for this are unclear. Immune responses to an oral lyophilized vaccine for animals have been documented with classical swine fever in swine [35]. Lyophilized BCG prepared for humans must be reconstituted with the diluent provided by the manufacturer for each specific vaccine [36]. Substituting diluents may make the vaccine ineffective. In the present study, modified Middlebrook’s 7H9 liquid media was used as a diluent. For cattle receiving lyophilized vaccine via the oral route, reconstitution would of necessity be with saliva, which may not be ideal. It is also not clear if sufficient reconstitution of vaccine occurred in the oral cavity or if reconstituted vaccine contacted oropharyngeal lymphoid tissues. Lack of contact between reconstituted vaccine and oropharyngeal lymphoid tissues may have precluded a measurable T cell proliferative response.

Consistent with the present study, previous studies have demonstrated that IFN-γ responses in orally vaccinated cattle are delayed compared to those of SQ vaccinated cattle with strong IFN-γ responses 8 weeks after vaccination and peaking at 11 weeks after vaccination [8]. The route of vaccine administration is known to affect the kinetics and quality of the subsequent immune response [37–39]. This is in part driven by antigen delivery and availability, which impacts priming of immune cells and the subsequent local and systemic immune responses. In general, intramuscular (IM) and SQ administration are the most used routes of vaccination. However, muscle is a relatively poor site for immune cell localization [40] as compared to the skin, which is a more immunocompetent site containing a wider array of antigen presenting cells (APC) including various subsets of dendritic cells (DC) that continually circulate between the skin, the draining lymph nodes, and peripheral blood [41]. Increased antigen and APC availability could lead to faster and more robust immune responses. Oral vaccine administration induces both mucosal and systemic immune responses [42]. As described earlier, mucosal tissues possess MALT, which are specialized lymphoid structures capable of antigen uptake and presentation to DC and macrophages. However, mucosal surfaces present a challenge to antigen delivery, as a vaccine must overcome environmental degradation from enzymes and pH as well as break through mucosal tolerance [43]. Moreover, there are challenges with oral vaccination of ruminants, such as rumen dilution factor, rumen microbial makeup, fermentation and fermentation products, increased levels of fatty acids, and increased retention time in the rumen, that are not relevant with monogastric animals [44, 45]. This is an area where more study is needed.

We hypothesize, that differences in the kinetics of the T cell immune responses observed between SQ and oral BCG vaccinates can be partially explained by decreased antigen availability (*i.e.* degradation of bacteria) as well as overcoming mucosal tolerance encountered in the oral mucosa. In support of this, we, and others, have previously demonstrated that a higher dose of BCG is needed during oral administration to achieve protection against *M. bovis* challenge as compared to SQ administration [7, 11, 46]. In addition to the kinetics and magnitude of the T cell immune response, other qualitative differences may be present, which warrant further study. For example, in other studies, variable numbers of orally vaccinated cattle were categorized as tuberculin skin test reactors [7, 8, 46] while most SQ vaccinated cattle produced positive tuberculin skin test results [7, 8, 46–48]. The reason for the absence of positive tuberculin skin test responses in vaccinated cattle in the current study is unclear. Most studies reporting tuberculin reactivity in BCG vaccinated cattle originate in the United Kingdom (UK) or New Zealand (NZ). In the present study the PPDs used were different than those used in the UK and NZ studies, as was the source of the BCG Danish vaccine. Additionally, most other studies in cattle have used Friesian or Friesian cross cattle, whereas the current study used Herefords. It is possible these variations account for differences in tuberculin reactivity in vaccinates. Similarly, studies in humans have shown strong IFN-γ responses in orally vaccinated humans, while the development of positive tuberculin skin test reactions was inconsistent [49].

Interestingly, but not surprisingly, we also observed a difference in the kinetics of the CD4 T cell response following vaccination between phase 1 and phase 2, particularly following SQ BCG vaccination at the 4-week time point. BCG has been extensively studied worldwide and its variability in protection ranges from 0 – 80%. This variability has been attributed to a variety of factors ranging from the host (genetics, nutrition, concurrent infections, health status) to the environment (sunlight exposure, seasons, infectious agents, other mycobacteria), and to the vaccine itself (cold chain preservation, genetic variability of strains, growth) (reviewed elsewhere [50, 51]). Here, variations in an animal’s individual genetic background are expected in an outbred population and would likely explain differences in kinetics. The animals used in both phases of this study originated from the same source and were housed in adjacent but separate barn spaces. However, when housing large animals outdoors we cannot predict or know what they may be exposed to, including environmental mycobacteria. These 2 phases were also conducted approximately 6 months apart and at different times of the year. Both seasonal variations and circadian rhythm have been shown to be affect trained immunity induced by BCG vaccination in humans [52, 53], factors which could also affect adaptive immune responses. It’s possible that all these variables together likely contributed to the observed differences. Another possible variable is the vaccine dose, which was similar between the two phases but administered at different times. From a statistical point of view, significance at individual time points was reached when both groups were analyzed together, which is expected given the variability observed even within animals in the same vaccine group and phase. This further highlights the need for larger groups of animals when performing studies in outbred populations, adding to the complexity of understanding host responses to vaccination.

*Mycobacterium bovis*, the cause of bovine TB is a fastidious, slow-growing, intracellular pathogen. As such, cell-mediated immune responses are necessary for protection, specifically, production of IFN-γ by CD4 T cells that activate macrophages to kill mycobacteria. Detection of IFN-γ produced by T cells is the most widely used method for monitoring immune responses following infection or vaccination [54]. However, increased IFN-γ production by T cells after vaccination is not an accurate predictor of vaccine-induced protection [55]. To be sure, identifying successful biomarkers of vaccine-induced protection for tuberculosis has been difficult and is an area of needed research.

## Conclusion

In summary, the present study demonstrated that oral vaccination with lyophilized BCG did not induce CD4 T cell proliferation or IFN-γ production by CD4 T cells in contrast to vaccination administered SQ or orally delivered liquid BCG. Although IFN-γ production by CD4 T cells is not a useful biomarker to evaluate vaccine-induced protection, it is useful as an indicator of vaccine processing by necessary lymphoid organs and vaccine-induced immune responses. Studies to assess vaccine efficacy are needed to determine if oral delivery of BCG provides protection against infection. Furthermore, oral delivery of BCG would be a practical means for mass vaccination, especially in wildlife species and if platforms are developed that allow for the passive delivery of vaccine. Current work in our laboratory is looking to develop such an approach.

## Methods

### Animals and vaccination

Twenty female Hereford heifers, 12–18 months of age, were purchased from a central Iowa, USA herd with no history of bovine tb. Due to limitations in barn space and technical assistance, vaccination was done in two phases. In phase one, 11 cattle were randomly divided into three groups. Group 1 (n = 4) received 5.4 × 10^6^ CFU BCG injected subcutaneously (SQ) in the lateral neck region, Group 2 (n = 4) received 3.1 × 10^7^ CFU liquid BCG, delivered to the posterior oral cavity as described previously [56]. Group 3 (n = 3) orally consumed 1.9 × 10^7^ CFU of lyophilized BCG contained within a pure gelatin capsule, size 000, placed within a small amount of a commercial alfalfa product (Chaffhaye; Dell City, TX, USA). In phase two, 9 animals were randomly divided into the same three groups were used. Group 1 (n = 3) received 6.3 × 10^5^ CFU BCG SQ, Group 2 (n = 3) received 3.2 × 10^7^ CFU liquid BCG orally in the posterior oral cavity, Group 3 (n = 3) orally consumed 2.0 × 10^7^ CFU lyophilized BCG within a gelatin capsule inside a small amount of Chaffhaye (Supplemental Table 1). All experimental animal procedures were conducted in accordance with recommendations in the Care and Use of Laboratory Animals by the National Institutes of Health and the Guide for the Care and Use of Agricultural Animals in Research and Teaching [57, 58]. Animal related procedures were also approved the USDA National Animal Disease Center Animal Care and Use Committee (protocol #ARS-2021–952).

*Mycobacterium bovis* BCG Danish 1331 was grown in Middlebrook’s 7H9 liquid media supplemented with 10% oleic-albumin-dextrose catalase (OADC) (Difco, Detroit, MI, USA) plus 0.05% Tween 80 (Sigma Chemical Co, St. Louis, MO, USA). Mid log-phase growth bacilli (21–28 days) were pelleted by centrifugation at 750 × g, resuspended in 7H9 media, and frozen at −80 °C in 1 ml aliquots until used. Frozen stock was warmed to room temperature (RT) and diluted to the appropriate cell density in 2 ml of 7H9 media. Bacilli were enumerated by serial dilution plate counting on Middlebrook’s 7H11 selective media (Becton Dickinson, Cockeysville, MD, USA) with 0.4% OADC.

### Lyophilization procedure

BCG Danish was washed twice with 1 ml World Health Organization (WHO) media to remove 7H9 growth media. WHO media is composed of Bacto™ Tryptone (Thermo Fisher, Waltham, MA, USA), sucrose, glutamic acid-L monosodium salt hydrate anhydrous and water.

After the second wash, 0.9 ml of WHO media was added to the cell pellet. Vials were sonicated 15 s (sec) in a sonicating water bath and subsequently vortexed for 20 s. Washed cell stock at a volume of 0.2 ml was then added to 5.2 ml of WHO media. This preparation was then aliquoted into sterile 2 ml glass serum vials (DWK Life Science, Millville, NJ, USA) at a volume of 0.2 ml. Lyophilization was done in a Millrock Magnum Freeze Dryer (Millrock Technology, Kingston, NY, USA) with 9, 60 min (min) freeze cycles at 10 °C, 5 °C, 0° C, −5 °C, −10 °C, 15 °C, −20 °C, −25 °C and −25 °C with a 180 min final freeze of −30 °C. Drying was done at a vacuum of −100 milliTorr (mTorr) with 7 cycles of 60 min at −25 °C, −20 °C, −15 °C, −10 °C, 0 °C, 10 °C, and 20 °C followed by 480 min at 22 °C. A secondary drying step was then done for 180 min at −25 °C. At that point the stoppers were placed in the vials and the vacuum released.

### Stability of lyophilized M. bovis* BCG*

Lyophilized BCG Danish was washed twice with 1 ml WHO media. After the second wash, 0.9 ml WHO media was added to the cell pellet. Vials were sonicated 15 s in a sonicating water bath and subsequently vortexed for 20 s. Washed cell stock at a volume of 0.2 ml was then added to 5.2 ml of 1) WHO media, 2) WHO media with lyoprotectants [1.5% (wt/vol) dextran/7.5% (wt/vol) lactose] or 3) WHO media with 0.04% (v/v) Tween® 80. All media were prepared by National Centers for Animal Health (NCAH) media preparation laboratory. Each of these media preparations were then aliquoted into sterile 2 ml glass serum vials (DWK Life Science, Millville, NJ, USA) at a volume of 0.2 ml as well as one additional vial of 0.3 ml to be used for before/after lyophilization colony counts. Serial 1:10 dilutions were completed on this marked vial from each of the three groups and 100 µl plated from each dilution onto 7H11 agar plates in duplicate. All vials were then lyophilized. After lyophilization, BCG aliquots were divided and stored at four different temperatures (33.8 °C, 25 °C, 4 °C or −20 °C). Marked vials used for dilution counts prior to lyophilization were selected and 0.2 ml 7H9/Tween® 80/OADC was added to reconstitute the BCG. Serial 1:10 dilutions were performed and 100 µl plated from each dilution onto 7H11 plates in duplicate to determine prelyophilization counts.

For the 33.8 °C temperature group, one vial was reconstituted with 0.2 ml 7H9/Tween® 80/OADC at 3 days, 7 days, 2 weeks, 1 month and 2 months after lyophilization. Contents were transferred to microfuge tubes and sonicated for 15 s and vortexed for 20 s. Serial 1:10 dilutions were completed and 100 µl from each dilution were plated in duplicate on 7H11 plates. For the 4 °C, 25 °C and −20 °C temperature groups, one vial was reconstituted with 0.2 ml 7H9/Tween® /OADC at 3, 6, 9, and 12 months. Contents were transferred to microfuge tubes and sonicated for 15 s and vortexed for 20 s. Serial 1:10 dilutions were completed and 100 µl from each dilution were plated in duplicate on 7H11 plates to obtain bacterial counts.

### Interferon Gamma Release Assay (IGRA)

Whole blood samples were collected in sodium-heparinized tubes (Becton, Dickinson and Company) from the jugular vein and transferred to the laboratory at RT within 2 h (h) of collection. In the laboratory, blood samples were stimulated within 10 h after collection. Samples were divided into four aliquots of 1.0 ml each in 48-well cell tissue culture plates (ThermoFisher). Purified protein derivative (PPD; ID Vet, Grabels, France) from *M. bovis* (PPD-B), purified protein derivative from *M. avium* (PPD-A), pokeweed mitogen (PWM; Thermo Fisher), and PBS as a negative control (nil) antigen were used for the stimulation of whole blood samples. One hundred microliters of PPD-B (0.3 μg/ml), PPD-A (0.3 μg/ml), PWM (positive control), and PBS (nil antigen) were added and mixed to individual wells and incubated at 37 °C with 5% CO_2_ for 16–24 h. After incubation, 48-well tissue culture plates were centrifuged for 20 min at 900 × g at 23 °C, and the upper layer of plasma was harvested. The samples were tested in duplicate using a sandwich enzyme immunoassay (Bovigam; Thermo-Fisher) as recommended by the manufacturer. The optical density (OD) of each well was measured at 450 nm with a 620– 650 nm reference filter. The mean OD of each sample was calculated and used to define the cutoff values. The OD of a sample stimulated with PPD-B minus the OD of a sample stimulated with PPD-A (OD_PPD−B_–OD_PPD−A_) (ΔOD) was used as cut-off criteria. When ΔOD was ≥ 0.1 the sample was considered positive.

### Peripheral blood mononuclear cell (PBMC) isolation

Whole blood was collected via jugular venipuncture and placed into tubes containing 2 × acid-citrate-dextrose (ACD) anticoagulant. PBMC were isolated via density centrifugation using Ficoll^â^ as previously described [59]. PBMC count and viability were determined via microscopic examination and counting using a hemocytometer and trypan blue exclusion. Cells were resuspended to a concentration of 1 × 10^7^ live cells per ml. To assess proliferation and intracellular cytokine production, PBMC were labeled with 1:10 CellTraceä Violet (eBioscience, Thermo Fisher) solution, according to manufacturer’s recommendations, and resuspended in complete RPMI media. PBMC were then plated, 1 × 10^6^ per well, onto 96-flat bottom plates, and left unstimulated, or stimulated with purified PPD-B (5 µg/well), or Concanavalin A (ConA; 0.5 µg/well; Sigma). Plates were incubated for 7 days at 37 °C with 5% CO_2_. To assess cytokine production, approximately 16 h prior to harvest, all wells were treated with a 1 × GolgiStop^TM^ solution (eBioscience) according to manufacturer’s recommendation.

### Flow cytometry staining and analysis

For surface and intracellular staining and flow cytometry, PBMC were prepared as described previously [59]. In brief, PBMC were transferred to 96-well round bottom plates, centrifuged at 300 × g at RT, and then washed in 1 × Dulbecco’s phosphate buffered saline (DPBS). PMBC were then incubated in a fixable cell viability dye solution (1:2000 dilution) (eBioscience) for 20 min at 4° C, and then washed once in 1 × DPBS and once in FACS buffer (0.5% fetal bovine serum (FBS) in PBS). PBMC were then resuspended with gentle vortexing, and incubated with anti-bovine γδ primary antibody (Washington State University, Pullman, WA) for 15 min at RT and then with anti-IgG2b fluorescently labeled secondary antibody for an additional 15 min at RT. PBMC were washed again twice in FACS buffer and incubated with a cocktail containing antibodies against bovine CD4 (FITC-labeled; CC30) and CD8 (AlexaFluor 647-labeled; CC58) (BioRad, Hercules, CA, USA) for 15 min at RT, followed by two additional washes in FACS buffer. For intracellular staining, PBMC were fixed and permeabilized using a permeabilization kit according to the manufacturer’s recommendations (Becton Dickinson Bioscience, Franklin Lakes, NJ, USA). Intracellular cytokine staining was performed using antibovine IFN-γ antibody (PE-labeled; CC302). PBMC were then washed twice in wash buffer (Becton Dickinson, Bioscience), once in FACS, and then resuspended in FACS buffer until analysis. Data was collected using the BD FACSymphony^TM^ flow cytometer (BD Bioscience) using the DIVA software and analyzed using FlowJo^®^ (Ashland, OR, USA).

### Tuberculin skin testing

The intradermal comparative cervical test (CCT) was administered prior to vaccination and 16 weeks post-vaccination according USDA guidelines. In brief, after the clipping of hair from two sites on the lateral neck, 100 µl (1.0 mg/ml) of PPD-B and 100 µl (0.4 mg/ml) of PPDA (National Veterinary Services Laboratories) were injected intradermally into separate sites on the lateral side of the neck. Seventy-two hours later, injection sites were evaluated visually, by palpation, and skin thickness measurements to the nearest millimeter using manual calipers. Change in skin thickness due to swelling or induration was calculated by subtracting the preinjection skin thickness from the postinjection skin thickness that was obtained 72 h after injection. Results were interpreted by plotting changes in skin thickness on a traditional CCT scattergram for bison and cattle (Form VS 6-22D, Veterinary Services, USDA; Supplemental Fig. 2).

### RNA isolation and cytokine RT-qPCR

Whole blood was collected into heparinized tubes (Becton Dickinson). Within 2 h from collection 1.0 ml of whole blood was distributed in 24-well plates with 100 µl of complete RPMI media spiked with PPD-B (20 µg/mL) and incubated overnight at 39 °C with 5% CO_2_. The following day, samples were diluted with 2.5 ml of Erythrocyte Lysis Buffer (EL Buffer, QIAGEN), vortexed, incubated on ice for 20 min. Samples were centrifuged 400 × g for 10 min at 4 °C and resulting pellets were washed and centrifuged again at 400 × g for 10 min at 4 °C.

Supernatants were removed and pellets were resuspended in 350 µl of RLT buffer and β-mercaptoethanol (Millipore Sigma) (10 µl/ml) and frozen at −80 °C until used. From frozen pellets RNA extraction was performed using the RNeasy Plus Mini kit (QIAGEN) per manufacturer’s instructions with additional QIAshredder column option (QIAGEN). Total RNA was used for cDNA synthesis using Superscript IV VILO Master Mix kit (Invitrogen) and manufacturer’s instructions. RT-qPCR was performed using QuantStudio 3 System (Thermo Fisher) to detect synthesized double strand DNAs. Reactions were performed with TaqMan Advanced master mix (Fisher Scientific) in a 20 µl reaction volume comprising 2 µl of cDNA. Probes for CXCL10, IL-21, and IL-13, and eukaryotic 18S rRNA control were utilized to manufacturer’s suggestions (Life Technologies, Thermo Fisher). For the relative cytokine expression at different timepoints, target gene cycle threshold (Ct) values for an individual animal’s data were normalized with internal control 18S rRNA using comparative 2^−ΔΔCT^ [60].

### Statistical analysis

For flow cytometry analysis, mean frequency of T cell subsets showing proliferation and IFN-γ production were graphed and analyzed using GraphPad Prism (GraphPad Software, Boston, MA, USA). For all analyses we independently evaluated phase 1, phase 2, and all combined data. Changes over time were evaluated by repeated measures with a mixed model. Specific contrasts of interest (within group compared to pre-vaccination or between vaccinate groups at various time points) were evaluated with an ANOVA fitting vaccination status (SQ, oral liquid or lyophilized BCG), time (weeks post-vaccination), and a (vaccination x time) interaction. Significance was determined when p-value ≤ 0.05; error bars represent standard errors.

For cytokine data, all analyses were performed in comparison to the pre-vaccination values for each animal, considered herein as the basal cytokine expression (relative expression of 1). Cytokine expression data was analyzed using a mixed linear model using the R statistical software. Effects of ‘Vaccination Route’ (liquid oral or SQ), Timepoint (weeks post vaccination), and the interaction of (Vaccination Route) x (Timepoint) were included in the model. Each cytokine was evaluated independently. Differences between specific contrasts of interest were compared using pairwise comparisons of Least Squares Means. Significance was determined when *p*-value ≤ 0.05, and error bars represent standard errors.

## Supplementary Information

Supplementary Material 1. Supplemental Table 1. (.docx) Routes of administration and dosages of *M. bovis* BCG in colony-forming units/ml. Supplemental Table 2. (.docx) Comparative cervical tuberculin skin test results of cattle vaccinated by various routes with *M. bovis* BCG Danish. Supplemental Table 3. (.docx) Survivability of BCG Danish pre-lyophilization and various times post-lyophilization while stored at 33.8°C. Supplemental Table 4. (.docx) Survivability of BCG Danish pre-lyophilization and various times post-lyophilization while stored at -20 °C, -4 °C, or 25 °C.

Supplementary Material 2. Supplemental Figure 1 (.pdf). Gating strategy for flow cytometry analysis. FSC = forward scatter; SSC = side scatter. Supplemental Figure 2 (.pdf). USDA approved scatterplot used for interpretation of CCT tuberculin skin test results.

## Data Availability

The original contributions presented in the study are included in the article/supplementary material, further inquiries can be directed to the corresponding author/s.

## Abbreviations

ACD: Acid citrate dextrose
APC: Antigen presenting cell
BCG: Bacillus Calmette-Guérin
CCT: Comparative cervical test
CD: Cluster of differentiation
CXCL: C-X-C motif chemokine ligand
DC: Dendritic cell
DPBS: Dulbecco’s phosphate buffered saline
FBS: Fetal bovine serum
FITC: Fluorescein isothiocyanate
IFN: Interferon
IGRA: Interferon gamma release assay
IL: Interleukin
PBMC: Peripheral blood mononuclear cells
PE: Phycoerythrin
PPD: Purified protein derivative
PWM: Pokeweed mitogen
RT-qPCR: Reverse transcription quantitative polymerase chain reaction
WHO: World health organization
