# Supplementary material for: Immune responses of cattle vaccinated by various routes with Mycobacterium bovis Bacillus Calmette-Guérin (BCG)
Source: BMC Vet Res. 2025 Jan 15;21:19. doi: 10.1186/s12917-024-04452-7 (PMC11734464; doi:10.1186/s12917-024-04452-7)
Supplement: Supplementary file 1 — Supplementary Material 1. Supplemental Table 1. (.docx) Routes of administration and dosages of M. bovis BCG in colony-forming units/ml. Supplemental Table 2. (.docx) Comparative cervical tuberculin skin test results of cattle vaccinated by various routes with M. bovis BCG Danish. Supplemental Table 3. (.docx) Survivability of BCG Danish pre-lyophilization and various times post-lyophilization while stored at 33.8°C. Supplemental Table 4. (.docx) Survivability of BCG Danish pre-lyophilization and various times post-lyophilization while stored at -20 °C, -4 °C, or 25 °C. [file 12917_2024_4452_MOESM1_ESM.zip › Supplemental Table 1 .docx]

**Supplemental Table 1**. Routes of administration and dosages of *M. bovis* BCG in colony-forming units/ml.

| **Route** | **Phase 1** | **Phase 2** |
| --- | --- | --- |
| SQ | 5.4 x 10^6^ | 6.3 x 10^5^ |
| Oral liquid | 3.1 x 10^7^ | 3.2 x 10^7^ |
| Oral lyophilized | 1.9 x 10^7^ | 2.0 x 10^7^ |
