# Supplementary material for: Immune responses of cattle vaccinated by various routes with Mycobacterium bovis Bacillus Calmette-Guérin (BCG)
Source: BMC Vet Res. 2025 Jan 15;21:19. doi: 10.1186/s12917-024-04452-7 (PMC11734464; doi:10.1186/s12917-024-04452-7)
Supplement: Supplementary file 1 — Supplementary Material 1. Supplemental Table 1. (.docx) Routes of administration and dosages of M. bovis BCG in colony-forming units/ml. Supplemental Table 2. (.docx) Comparative cervical tuberculin skin test results of cattle vaccinated by various routes with M. bovis BCG Danish. Supplemental Table 3. (.docx) Survivability of BCG Danish pre-lyophilization and various times post-lyophilization while stored at 33.8°C. Supplemental Table 4. (.docx) Survivability of BCG Danish pre-lyophilization and various times post-lyophilization while stored at -20 °C, -4 °C, or 25 °C. [file 12917_2024_4452_MOESM1_ESM.zip › Supplemental Table 2.docx]

**Supplemental Table 2.** Comparative cervical tuberculin skin test results of cattle vaccinated by various routes with *M. bovis* BCG Danish. Values in parentheses represent change in skin thickness (to nearest 0.5 mm) at the PPD injection sites (*M. avium* PPD/*M. bovis* PPD). Status (reactor, suspect, negative) determined using Supplemental Figure 3.

| Animal | Group | Vaccine | Pre-Vaccination | 16 wks post-vaccination |
| --- | --- | --- | --- | --- |
| 504 | 1 | Liquid | Suspect (1.5/5.0) | Negative (3.5/3.0) |
| 510 | 1 | Liquid | Suspect (0.5/3.5) | Negative (2.0/0.5) |
| 513 | 1 | Liquid | Negative (2.5/3.0) | Negative (1.0/0.0) |
| 520 | 1 | Liquid | Negative (4.5/0.0) | Negative (2.0/2.5) |
| 511 | 1 | Lyophilized | Negative (0.0/0.0) | Negative (3.5/0.0) |
| 515 | 1 | Lyophilized | Negative (0.0/0.0) | Negative (0.5/0.0) |
| 519 | 1 | Lyophilized | Negative (1.5/2.5) | Negative (1.0/0.5) |
| 512 | 1 | SQ | Negative (3.0/0.0) | Negative (0.0/0.0) |
| 518 | 1 | SQ | Negative (4.5/0.5) | Negative (0.5/2.0) |
| 522 | 1 | SQ | Negative (3.0/0.0) | Suspect (0.5/4.0) |
| 524 | 1 | SQ | Negative (1.0/0.0) | Negative (1.5/3.0) |
| 505 | 2 | Liquid | Suspect (4.5/6.5) | Negative (4.0/4.0) |
| 506 | 2 | Liquid | Negative (2.5/2.5) | ND |
| 507 | 2 | Liquid | Negative (2.5/1.0) | Negative (1.0/0.0) |
| 508 | 2 | Lyophilized | ND | ND |
| 514 | 2 | Lyophilized | Suspect (4.0/7.0) | Negative (4.0/2.0) |
| 523 | 2 | Lyophilized | Negative (0.0/0.0) | Negative (0.0/0.0) |
| 502 | 2 | SQ | Negative (0.5/0.0) | ND |
| 509 | 2 | SQ | Negative (4.0/2.0) | Negative (3.0/2.0) |
| 517 | 2 | SQ | Negative (4.0/2.5) | Negative (3.5/2.5) |

Liquid= liquid instilled in posterior oral cavity.

Lyophilized= lyophilized BCG within a gelatin capsule contained within Chaffhaye.

SQ= BCG administered subcutaneously in lateral neck region.

NV= non-vaccinated

ND= not done
