# Supplementary material for: Immune responses of cattle vaccinated by various routes with Mycobacterium bovis Bacillus Calmette-Guérin (BCG)
Source: BMC Vet Res. 2025 Jan 15;21:19. doi: 10.1186/s12917-024-04452-7 (PMC11734464; doi:10.1186/s12917-024-04452-7)
Supplement: Supplementary file 1 — Supplementary Material 1. Supplemental Table 1. (.docx) Routes of administration and dosages of M. bovis BCG in colony-forming units/ml. Supplemental Table 2. (.docx) Comparative cervical tuberculin skin test results of cattle vaccinated by various routes with M. bovis BCG Danish. Supplemental Table 3. (.docx) Survivability of BCG Danish pre-lyophilization and various times post-lyophilization while stored at 33.8°C. Supplemental Table 4. (.docx) Survivability of BCG Danish pre-lyophilization and various times post-lyophilization while stored at -20 °C, -4 °C, or 25 °C. [file 12917_2024_4452_MOESM1_ESM.zip › Supplemental Tables 3 and 4.docx]

**Supplemental Table 3. Survivability of BCG Danish pre-lyophilization and various times post-lyophilization while stored at 33.8°C.**

| Pre-lyophilization | Post-lyophilization | 3 days | 7 days | 2 weeks | 1 month | 2 months |
| --- | --- | --- | --- | --- | --- | --- |
| 6.4 x10^7^ CFU^a^/ml | 1.3 x10^7^ CFU/ml | 8.9 x10^6^ CFU/ml | 7.6 X 10^6^ CFU/ml | 2.45 x 10^6^ CFU/ml | 7.35 x 10^5^ CFU/ml | 2.08 x 10^5^ CFU/ml |

^a^Colony-forming units.

**Supplemental Table 4. Survivability of BCG Danish pre-lyophilization and various times post-lyophilization while stored at -20 °C, -4 °C, or 25 °C.**

| Storage Temperature | Pre-lyophilization | Post-lyophilization | 3 months | 6 months | 9 months | 12 months |
| --- | --- | --- | --- | --- | --- | --- |
| -20 °C | 6.4 x10^7^ CFU^a^/ml | 1.3 x10^7^ CFU/ml | 9.65 x 10^6^ CFU/ml | 1.24 x 10^7^ CFU/ml | 1.45 x 10^7^ CFU/ml | 1.06 x 10^7^ CFU/ml |
| -4 °C | 6.4 x10^7^ CFU^a^/ml | 1.3 x10^7^ CFU/ml | 7.05 x 10^6^ CFU/ml | 6.2 x 10^6^ CFU/ml | 7.4 x 10^6^ CFU/ml | 1.57 x 10^6^ CFU/ml |
| 25 °C | 6.4 x10^7^ CFU^a^/ml | 1.3 x10^7^ CFU/ml | 1.56 x 10^6^ CFU/ml | 1.67 x 10^5^ CFU/ml | 1.35 x 10^5^ CFU/ml | 3.2 x 10^4^ CFU/ml |

^a^Colony-forming units.
