# Supplementary material for: Immune responses of cattle vaccinated by various routes with Mycobacterium bovis Bacillus Calmette-Guérin (BCG)
Source: BMC Vet Res. 2025 Jan 15;21:19. doi: 10.1186/s12917-024-04452-7 (PMC11734464; doi:10.1186/s12917-024-04452-7)
Supplement: Supplementary file 2 — Supplementary Material 2. Supplemental Figure 1 (.pdf). Gating strategy for flow cytometry analysis. FSC = forward scatter; SSC = side scatter. Supplemental Figure 2 (.pdf). USDA approved scatterplot used for interpretation of CCT tuberculin skin test results. [file 12917_2024_4452_MOESM2_ESM.zip › Supplemental Figure 2.pdf]

USDA - APHIS - VETERINARY SERVICES

FORM APPROVED - OMB NO. 0579-0146 EXP. X/XX

COMPARATIVE CERVICAL TUBERCULIN TEST RESULTS ☐ BOVINE ☐ CERVINE OR ☐ OTHER ____________

NAME OF HERD OWNER *(Last, First, Middle Initial)*	ADDRESS *(Including Zip Code)*

COMPARATIVE TEST		PRIOR CFT OR SCT		
NUMBER TESTED	DATE INJECTED	NUMBER TESTED	DATE INJECTED	
COMPARATIVE RETEST ☐ 1ST ☐ 2ND ☐ 3RD	OBSERVATION DATE	NEG.	SUS.	REA.

NAME OF VETERINARIAN	TITLE	DATE

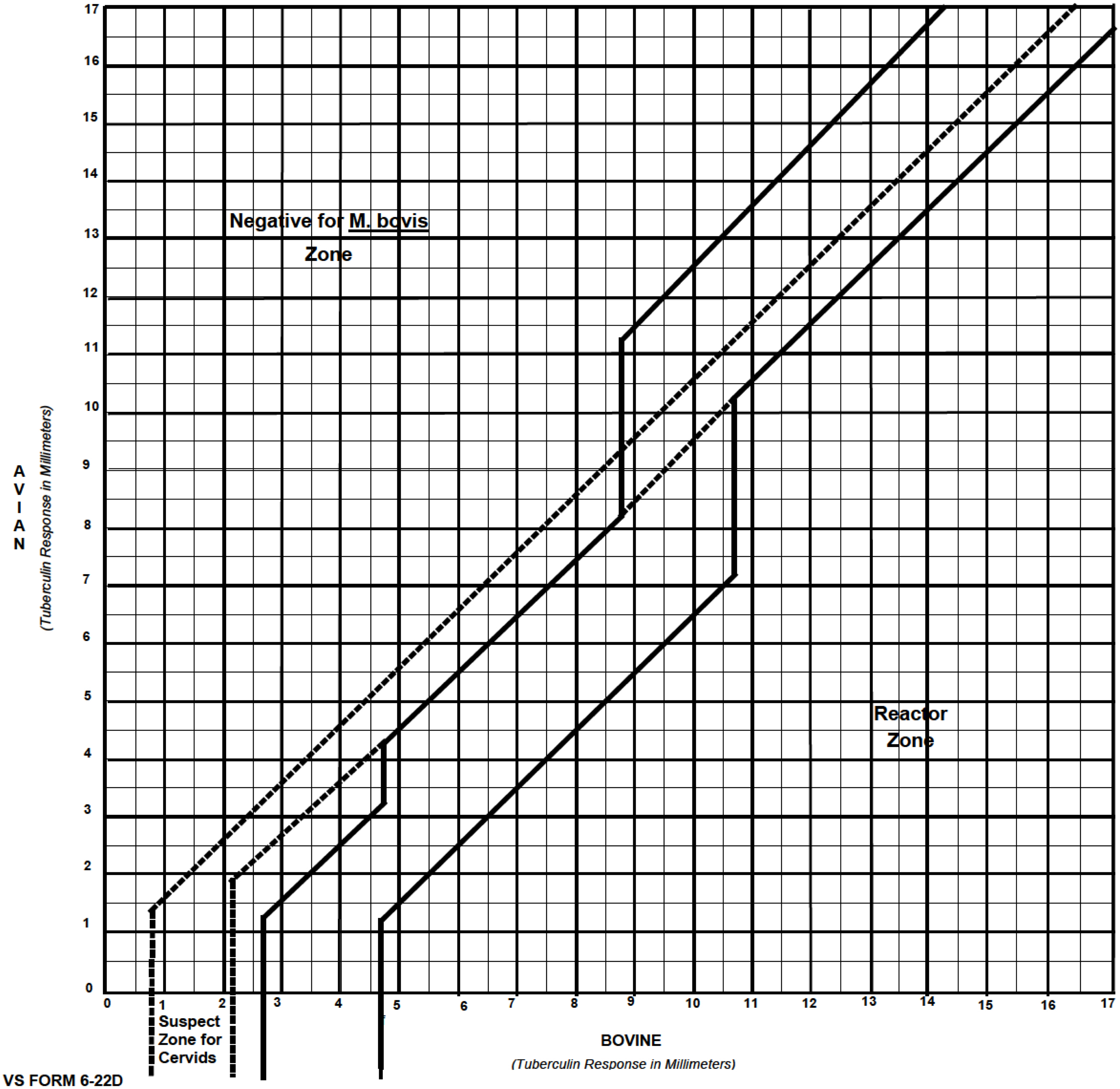

VS FORM 6-22D
(FEB 99)

(Previous editions are obsolete.)
