# Supplementary figures and images for: Immune responses of cattle vaccinated by various routes with Mycobacterium bovis Bacillus Calmette-Guérin (BCG)
Source: BMC Vet Res. 2025 Jan 15;21:19. doi: 10.1186/s12917-024-04452-7 (PMC11734464; doi:10.1186/s12917-024-04452-7)

# Supplementary Figure 1. Gating strategy for flow cytometry analysis

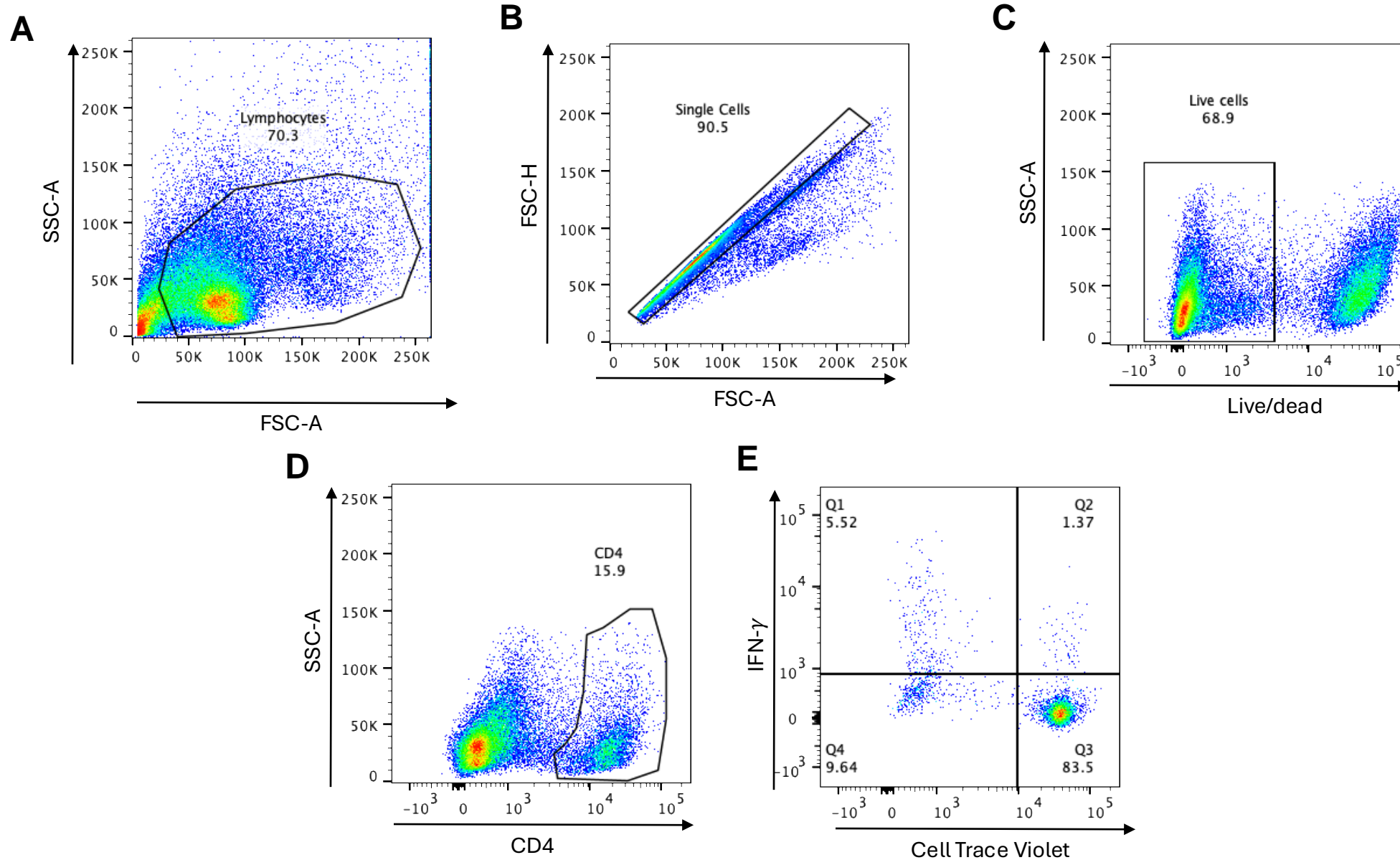

Supplement: Supplementary file 2 — Supplementary Material 2. Supplemental Figure 1 (.pdf). Gating strategy for flow cytometry analysis. FSC = forward scatter; SSC = side scatter. Supplemental Figure 2 (.pdf). USDA approved scatterplot used for interpretation of CCT tuberculin skin test results. [file 12917_2024_4452_MOESM2_ESM.zip › Supplementary Figure 1.pdf]
